# Mindfulness teacher training enhances interoceptive awareness and reduces emotional distress: a controlled study

**DOI:** 10.3389/fpsyg.2025.1488204

**Published:** 2025-04-25

**Authors:** Alberto Chiesa, Cristiano Crescentini, Fabio D’Antoni, Alessio Matiz

**Affiliations:** ^1^Istituto Mente Corpo, Bologna, Italy; ^2^Scuola Di Psicoterapia Cognitiva, Roma, Italy; ^3^Department of Languages and Literatures, Communication, Education and Society, University of Udine, Udine, Italy; ^4^School of Advanced Studies Sant’Anna, Institute of Mechanical Intelligence, Pisa, Italy; ^5^Maternal Infant Services Unit of Udine, Azienda Sanitaria Universitaria Friuli Centrale (ASUFC), Udine, Italy; ^6^Department of Psychology, Sapienza University of Rome, Rome, Italy

**Keywords:** mindfulness teachers, mindfulness teacher training, interoception, mind-body integration, anxiety, depression

## Abstract

**Introduction:**

Several mindfulness-based interventions (MBIs) have shown efficacy in enhancing interoceptive awareness (IA), the ability to perceive and interpret bodily signals, leading to improved mental and physical wellbeing. However, no study has yet explored the effects of mindfulness practice on IA in individuals training to become MBI teachers. Thus, we investigated the impact of a mindfulness teacher training (MTT) program on emotional distress and IA in individuals training to become mindfulness teachers.

**Methods:**

A group of 38 individuals undergoing MTT and a control group of 24 matched individuals were assessed before (T0) and after (T1) the 9 months MTT program. Emotional distress was assessed through the Hospital Anxiety and Depression Scale (HADS) and IA was assessed through the Multidimensional Assessment of Interoceptive Awareness (MAIA).

**Results:**

The MTT group showed significantly higher increases in the awareness of mind-body integration in comparison with the control group. Although no significant between-group changes were observed in emotional distress, increases in MAIA self-regulation scores within the MTT group were associated with decreases in HADS depression and total emotional distress scores.

**Discussion:**

This study offers further support to the positive impact of mindfulness practice on IA within an MTT program, suggesting that mindfulness training for future MBI teachers further enhances their ability to attend to and to regulate and interpret bodily signals. Future research should investigate the long-term impact of mindfulness training on IA and on mental health in comparison with active comparators.

## 1 Introduction

The complex interplay between mental and physical processes has long been a subject of scientific exploration (James, 1890). The integration of Western and Eastern perspectives has fostered renewed interest in this dynamic, particularly within the field of mindfulness research (Khoury et al., 2017). Mindfulness, originating from contemplative traditions, has gained significant interest in both clinical psychology and neuroscience studies for its potential to improve mental and physical health (Creswell, 2017).

The cultivation of interoceptive awareness (IA), defined as the ability to perceive and interpret internal bodily signals, has been proposed as a core component of mindfulness practice (Khoury et al., 2017). Converging evidence from various studies suggests that mindfulness-based interventions (MBIs) can effectively enhance specific dimensions of IA and that, in turn, these enhancements are associated with a range of positive outcomes for mental and physical health. Such outcomes have been observed, for instance, in terms of improvements in anxiety and depression levels (de Jong et al., 2016; Fissler et al., 2016; Matiz et al., 2020a), dissociative tendencies (D’Antoni et al., 2022), unhealthy eating patterns (Ugarte Pérez et al., 2023), substance craving (Price et al., 2019), therapeutic adherence (Loucks et al., 2023), and cognitive functioning (Galluzzi et al., 2024). Many of these studies employed the Multidimensional Assessment of Interoceptive Awareness (MAIA) questionnaire, a well-validated and frequently used tool that provides a comprehensive assessment of various dimensions of IA (Mehling et al., 2012). Such multidimensional instruments are needed due to the multifaceted nature of IA, which encompasses both physiological awareness and evaluative interpretations of bodily signals, and can offer a significant pathway for understanding the mechanisms through which mindfulness exerts its beneficial effects on health (Khoury et al., 2017).

Despite the body of research on MBI effects on IA, no study has yet investigated the extent to which mindfulness teacher training (MTT) programs can enhance IA in future MBI teachers. MTT is supposed to be delivered after participants have completed an introductory 8 weeks MBI and subsequently practiced some amount of personal mindfulness meditation. Future mindfulness teachers should thus have developed their mindfulness and IA skills before the MTT, and should continue to cultivate them during the MTT itself. In the context of MBIs, the development of IA skills has been linked to the process of embodiment of mindfulness (Khoury et al., 2017; Hanley et al., 2017). In this regard, theoretical contributions and guidelines regarding the structure and content of MTTs have emphasized the teacher’s personal embodiment of mindfulness, alongside other professional competences such as relational skills and proficiency in guiding mindfulness practices, as central to an effective delivery and participant outcomes (e.g., Crane et al., 2010; Crane et al., 2012; Crane et al., 2020; Griffith et al., 2021). The importance of embodying mindfulness to be effective as an MBI teacher, primarily due to the need to serve as role models for MBI participants, has also emerged from individual and group reports of mindfulness teachers and trainee teachers (van Aalderen et al., 2014; Bowden et al., 2021).

However, the assessment of IA as an expression of embodied mindfulness in future mindfulness teachers has not yet been taken into account. In general, research on the effects of MTTs on future mindfulness teachers is scarce. Two studies have explored the experiences of mindfulness teacher trainees during the training process using qualitative and quantitative self-reports, revealing trainees’ feelings during the training and satisfaction with it, as well as perceived strengths and limits of the program (Marx et al., 2015; Fontana et al., 2024). Only two studies have utilized validated questionnaires to assess the changes that occur during MTT, highlighting that these changes were associated with improved mindfulness skills, emotion regulation, and psychological wellbeing (Crane et al., 2020; Matiz et al., 2025).

Therefore, building on the existing literature on the MBI effects on IA, the present study aims to further elucidate the extent to which mindfulness practice could enhance IA in the context of a 9 months MTT. A group of control participants was employed alongside the group of mindfulness teacher trainees. The MAIA questionnaire was chosen to measure IA because of its multidimensional nature and its suitability for assessing IA within the context of MBIs. Due to the paucity of studies on MTT effects in comparison to those on MBI effects, and the differences between MBIs and MTTs (in terms of duration, content and structure), as well as between MBI and MTT participants (in terms of general interest and previous experience in mindfulness, and possibly higher levels of baseline mindfulness-related variables), no specific hypotheses were formulated about which dimensions of IA could be affected by MTT. Moreover, due to the general association between mindfulness training and emotion regulation, the study also aims to evaluate the extent to which IA could be related to emotional distress of the sample of mindfulness teachers trainees.

## 2 Materials and methods

### 2.1 Participants

The present research focused on individuals participating in an MTT to become mindfulness teachers (*n* = 60). Those who were available to participate and completed the study formed the MTT group (*n* = 38). Study participants were medical doctors or psychologists, some of whom were psychotherapists or psychotherapy trainees as well. They ranged in age from 24 to 68 and did not have psychiatric disorders.

**FIGURE 1:**
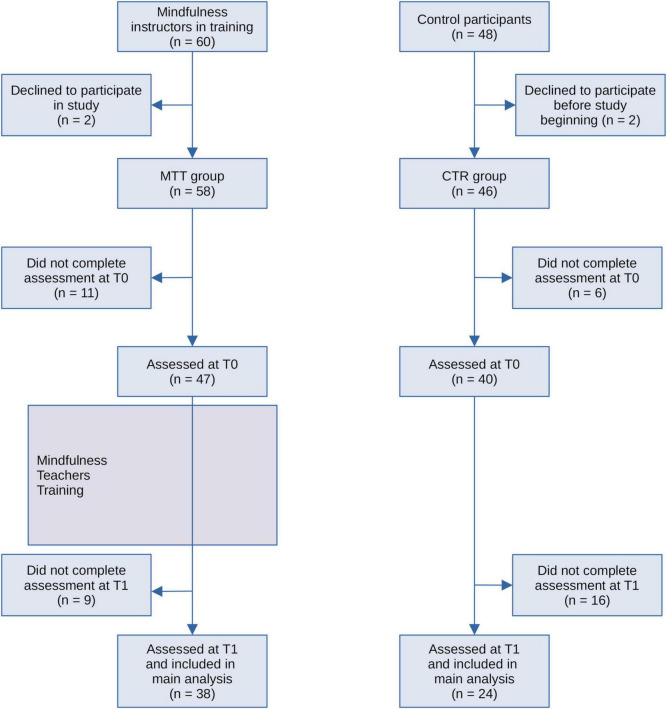
The participants flow chart diagram. CTR, control, MTT, mindfulness teacher training. T0, before the MTT; T1, at the conclusion of the 9 months MTT.

The study also included a group of control participants (CTR group). They were recruited by MTT participants, who were required to find a familiar individual without any significant health issues, matching their gender, age and educational background, but not participating in mindfulness training neither undergoing any mindfulness practice. Most MTT participants were able to engage a control subject that met these criteria (*n* = 48), 24 of whom completed questionnaires about emotional distress and IA (see Figure 1).

The drop-out rate in study participation was not due to withdrawals from the MTT program (all MTT trainees completed the program); rather, it was due to the non-specific unavailability of some MTT participants to fill in the questionnaires and/or to contribute the questionnaires from their control participant.

### 2.2 Measures

#### 2.2.1 Emotional distress

Emotional distress was assessed with the Hospital Anxiety and Depression Scale (HADS; Zigmond and Snaith, 1983), in its Italian version (Costantini et al., 1999). This instrument consists of 14 items and utilizes a Likert scale response format with four levels (that vary depending on the specific item). It provides separate scores for Anxiety (seven items), Depression (seven items) and Total emotional distress. In the present study, Cronbach’s α for HADS Anxiety, Depression and Total emotional distress were 0.83, 0.74, and 0.86, respectively.

#### 2.2.2 Interoceptive awareness

Interoceptive awareness was assessed with the Multidimensional Assessment of Interoceptive Awareness questionnaire (MAIA; Mehling et al., 2012), in its Italian version (Calì et al., 2015). This instrument consists of 32 items and utilizes a Likert scale response format with six levels (from 0 = never, to 5 = always). It provides separate scores for eight subscales (Mehling et al., 2012): Noticing (“awareness of uncomfortable, comfortable, and neutral body sensations,” four items), Not Distracting (“tendency not to ignore or distract oneself from sensations of pain or discomfort,” five items), Not Worrying (“tendency not to worry or experience emotional distress with sensations of pain or discomfort,” three items), Attention Regulation (“ability to sustain and control attention to body sensations,” seven items), Emotional Awareness (“awareness of the connection between body sensations and emotional states,” five items), Self-Regulation (“ability to regulate distress by attention to body sensations,” four items), Body Listening (“active listening to the body for insight,” three items), and Trusting (“experience of one’s body as safe and trustworthy,” three items).

As reported in the development study of the MAIA (Mehling et al., 2012; see also Fissler et al. 2016), these eight subscales reflect five overall dimensions, as the Not Distracting and Not Worrying subscales form the dimension of Emotional Reactions and Attentional Response to a Sensation, while Emotional Awareness, Self-Regulation and Body Listening form the dimension of Awareness of Mind-Body Integration. In the present study, Cronbach’s α for the five MAIA dimensions, i.e., (1) Noticing, (2) Emotional Reactions and Attentional Response to a Sensation, (3) Attention Regulation, (4) Awareness of Mind-Body Integration and (5) Trusting, were 0.82, 0.51, 0.91, 0.94, and 0.88, respectively.

### 2.3 Procedure

Three MTT courses were conducted over a 4 years period (2019–2022). For each run of the MTT program, participants of both MTT and CTR groups were assessed in the week before (Time T0) and in the week following the end of the MTT program (Time T1) with paper questionnaires.

The MTT program lasted 9 months. The structure and content of the three runs of the MTT program were identical. The MTT program involved for 3 days residential retreats at the hermitage of Monte Giove (Fano, Italy), where participants received training, accommodation and meals. After the initial retreat, the other retreats were held approximately 2, 5, and 9 months later. Each retreat consisted of at least 16 h of mindfulness meditation practice, 5 h of lectures, and 4 h of supervised mindfulness practice. The following topics were covered during the lectures: foundations of Buddhist psychology, elements and applications of Mindfulness-Based Stress Reduction (MBSR; Kabat-Zinn, 1990) and Mindfulness-Based Cognitive Therapy (MBCT; Segal et al., 2002), trainer attitudes, how to guide the inquiry, fundamentals of Buddhist psychology, clinical and neuroscientific underpinnings of MBIs, and guidance on the technical and attitudinal facets of leading MBSR and MBCT groups. The MTT program was led by one of the authors (AC), a medical doctor specialized in psychiatry and psychotherapy with extensive experience as a meditation practitioner, certified mindfulness teacher and instructor of mindfulness teachers for over 10 years.

### 2.4 Statistical analysis

The statistical analysis was performed with R version 3.6.3.

Missing responses within each participant’s questionnaire were initially examined, individually for the HADS and MAIA questionnaires. If the number of missing responses in a participant’s questionnaire reached/exceeded 30% of the total number of items, that particular participant’s questionnaire was excluded from further analysis; alternatively, the missing values were replaced by the nearest response level to the average score of the corresponding group for the respective item. Using this criterion, six participants (five from the MBI group and one from the CTR group) were excluded due to partial completion of questionnaires at baseline, and three participants (one from the MBI group and two from the CTR group) were excluded at study completion. Among study completers (Figure 1), the percentage of imputed values for missing responses on the total number of responses was 0.86%.

Participants with HADS and MAIA scores at baseline (T0) and at study completion (T1) were included in the main analysis of the study. These concerned a series of 2 × 2 mixed-model analyses of variance (ANOVAs) on scores from the HADS and MAIA questionnaires. These analyses examined the effects of the “Group” variable (with levels: MBI, CTR) as a between-subject factor, and the “Time” variable (with levels: T0, T1) as a within-subject factor. However, due to significant deviations from normality in the within-group distribution of the HADS and MAIA scores, as determined through the Shapiro-Wilk normality test, robust ANOVAs on trimmed means were utilized. These robust ANOVAs supply Q statistics and p values for the main effects of Group and Time, as well as for the Group × Time interaction. The trimming level was set at the default value of 20%. *Post-hoc* pairwise comparisons were performed using the Holm-Bonferroni procedure. Additional analysis concerned the relationship between the T1−T0 change of HADS and MAIA scores within the MBI group, which was investigated by means of Spearman bivariate correlations. All effects are reported as significant at *p* < 0.05.

## 3 Results

### 3.1 Baseline characteristics of participants

After screening for missing or incomplete questionnaire entries, the valid retained questionnaires were 62 (females/males: 42/20): the MTT group comprised 38 participants (females/males: 25/13), and the CTR group 24 participants (females/males: 17/7) (see Figure 1). The MTT and CTR groups were not different in terms of baseline levels of emotional distress, or interoceptive awareness (|t| ≤ 1.9, *p* ≥ 0.064).

No significant baseline differences were found between the 62 participants who were included in the main analysis of the study (i.e., those with HADS and MAIA scores at both assessment time points) and the 25 participants who were lost after the baseline assessment, in terms of HADS (for all subscales, |t| < 0.8, *p* > 0.421) and MAIA (for all dimensions, |t| < 1.4, *p* > 0.166) scores.

### 3.2 Intervention effects

Scores in the two experimental groups at baseline (Time = T0) and study completion (Time = T1) are summarized in Table 1 and intervention effects in Table 2.

**TABLE 1:** Scores in the two experimental groups at baseline (Time = T0) and study completion (Time = T1).

	M (SD) in the MTT group (*n* = 38)		M (SD) in the CTR group (*n* = 24)	
**Scale**	**Time = T0**	**Time = T1**	**Time = T0**	**Time = T1**
HADS anxiety	5.8 (3.1)	4.5 (3.2)	7.0 (3.0)	6.8 (3.5)
HADS depression	2.7 (1.6)	2.1 (1.8)	3.9 (3.3)	3.1 (3.6)
HADS total (anxiety + depression)	8.5 (4.1)	6.6 (4.4)	11.0 (5.5)	9.9 (6.7)
MAIA 1) Noticing	13.9 (3.3)	15.9 (2.0)	11.8 (4.9)	12.8 (3.4)
MAIA 2) Emotional reactions and attentional response to a sensation (not distracting + not worrying)	15.3 (2.5)	13.8 (3.3)	16.7 (3.9)	15.0 (3.0)
MAIA 3) Attention regulation	21.0 (5.6)	25.4 (3.4)	18.4 (7.6)	18.8 (8.4)
MAIA 4) Awareness of mind-body integration (emotional awareness + self-regulation + body listening)	39.2 (9.1)	46.5 (6.1)	34.0 (13.2)	34.6 (13.0)
MAIA 5) Trusting	10.4 (3.1)	12.0 (2.0)	10.3 (3.3)	9.8 (3.5)

CTR, control; HADS, Hospital Anxiety and Depression Scale; M, mean, MAIA, Multidimensional Assessment of Interoceptive Awareness; MTT, mindfulness teacher training; SD, standard deviation.

**TABLE 2:** Results of the robust ANOVAs on trimmed means.

	Group effect		Time effect		Group × Time effect	
**Scale**	**Q**	**p**	**Q**	**p**	**Q**	**p**
HADS anxiety	6.0	0.018*	3.1	0.087	1.6	0.208
HADS depression	1.0	0.336	6.2	0.018*	0.6	0.436
HADS total (anxiety + depression)	4.3	0.046*	10.0	0.003**	0.9	0.361
MAIA 1) Noticing	12.5	0.001**	3.1	0.091	1.4	0.251
MAIA 2) Emotional reactions and attentional response to a sensation (not distracting + not worrying)	2.6	0.113	7.5	0.009**	0.2	0.648
MAIA 3) Attention regulation	7.5	0.010*	7.8	0.008**	2.8	0.103
MAIA 4) Awareness of mind-body integration (emotional awareness + self-regulation + body listening)	8.1	0.008**	5.5	0.026*	5.5	0.026*
MAIA 5) Trusting	1.3	0.271	1.0	0.328	4.0	0.053

HADS, Hospital Anxiety and Depression Scale; MAIA, Multidimensional Assessment of Interoceptive Awareness,

*p < 0.05,

**p < 0.01.

#### 3.2.1 Emotional distress

In the HADS Anxiety score, a main effect of Group was observed (MTT < CTR; Q = 6.0, *p* = 0.018). In the HADS Depression score, a main effect of Time was observed (T0 > T1; Q = 6.2, *p* = 0.018). In the HADS Total emotional distress score, both a main effect of Group (MTT < CTR; Q = 4.3, *p* = 0.046) and of Time (T0 > T1; Q = 10.0, *p* = 0.003) were observed. No other differences emerged as significant (for all, Q < 3.1, *p* > 0.087).

#### 3.2.2 Interoceptive awareness

In the MAIA dimension of Noticing, a main effect of Group was observed (MTT > CTR; Q = 12.5, *p* = 0.001). In the MAIA dimension of Emotional Reactions and Attentional Response to a Sensation, a main effect of Time was observed (T0 > T1; Q = 7.5, *p* = 0.009). In the MAIA dimension of Attention Regulation, both a main effect of Group (MTT > CTR; Q = 7.5, *p* = 0.010) and of Time (T0 < T1; Q = 7.8, *p* = 0.008) were observed. In the MAIA dimension of Awareness of Mind-Body Integration, both a main effect of Group (MTT > CTR; Q = 8.1, *p* = 0.008) and of Time (T0 < T1; Q = 5.5, *p* = 0.026) were observed, as well as a Group × Time interaction effect (Q = 5.5, *p* = 0.026): scores improved significantly from T0 to T1 in the MTT group [t(37) = −6.5, *p* < 0.001], but did not change in the CTR group [t(23) = −0.2, *p* = 0.819]. Finally, in the MAIA dimension of Trusting, no significant effect was found (for all, Q < 4.0, *p* > 0.053). No other differences emerged as significant (for all, Q < 3.1, *p* > 0.087).

### 3.3 Correlations

Overall, no significant correlations were observed, within the MTT group, between the T1−T0 (post−pre intervention) change of HADS and MAIA scores (see Table 3). However, a significant relationship emerged as significant between the change score in MAIA Self-Regulation, which is a component of the MAIA dimension of Awareness of Mind-Body Integration, and the change in HADS Depression (r = −0.32, *p* = 0.048) and HADS Total emotional distress scores (r = −0.33, *p* = 0.044).

**TABLE 3:** Correlations between the T1–T0 change of HADS and MAIA scores within the MTT group.

Scale	HADS anxiety	HADS depression	HADS total
MAIA 1) Noticing	−0.05	−0.30	−0.22
MAIA 2) Emotional reactions and attentional response to a sensation	0.05	0.07	0.05
MAIA 2) component: MAIA not distracting	0.08	−0.07	0.01
MAIA 2) component: MAIA not worrying	0.06	0.29	0.16
MAIA 3) Attention regulation	−0.18	−0.17	−0.21
MAIA 4) Awareness of mind-body integration	−0.22	−0.15	−0.25
MAIA 4) component: MAIA emotional awareness	−0.20	−0.08	−0.15
MAIA 4) component: MAIA self-regulation	−0.19	−0.32*	−0.33*
MAIA 4) component: MAIA body listening	−0.16	−0.06	−0.17
MAIA 5) Trusting	−0.21	−0.17	−0.22

HADS, Hospital Anxiety and Depression Scale; MAIA, Multidimensional Assessment of Interoceptive Awareness; MTT, mindfulness teacher training,

*p < 0.05.

## 4 Discussion

The current study aimed at investigating the impact of an MTT program on IA and emotional distress in individuals participating in the training in comparison with a group of individuals not undergoing the training. The results showed that participants in the MTT program reported significantly higher increases in the “awareness of mind-body integration,” as measured with the MAIA, compared to the control group. Furthermore, within the MTT group, increases in MAIA self-regulation scores, which is a component of the MAIA dimension “awareness of mind-body integration,” were associated with decreases in HADS depression and total emotional distress scores.

The present study focused on mindfulness training for future mindfulness teachers, which is a topic rarely addressed in the literature. Indeed, beyond some valuable theoretical contributions and proposed international guidelines (e.g., Crane et al., 2010, 2012, 2020; Shonin and Van Gordon, 2015; Griffith et al., 2021; Kenny et al., 2020), only two studies have evaluated the pre-to-post training effects of MTT on participants (Crane et al., 2020; Matiz et al., 2025). Although the proposed guidelines on teaching assessment criteria for MBIs developed by Crane et al. (2013) highlighted the importance of embodying mindfulness in mindfulness teachers during their MTT, and various reports of mindfulness teachers and mindfulness trainee teachers confirmed this view (van Aalderen et al., 2014; Bowden et al., 2021), no previous study has evaluated the pre-to-post MTT effects on IA. Past research was limited to the effects of MTT on mindfulness skills, emotion regulation, and psychological wellbeing (Crane et al., 2020; Matiz et al., 2025). However, despite the differences in duration, content, and structure between MBIs and MTTs, as well as between the participants in these two trainings (such as their general interest and prior experience in mindfulness, along with potentially higher baseline levels of mindfulness-related variables), the findings of the present study can still be meaningfully compared to research examining the effects of introductory MBIs on IA.

On the one hand our findings confirm the general trend of MBIs improving IA, on the other it is important to underscore some key distinctions as well. First of all, in line with our study, several studies found that one or more dimensions of the “awareness of mind-body integration,” such as self-regulation, body listening, and emotional awareness improved following different mindfulness trainings both in comparison with active and inactive control groups (de Jong et al., 2016; Price et al., 2019; Matiz et al., 2020a,b; Lima-Araujo et al., 2022; D’Antoni et al., 2022; Feruglio et al., 2023; Loucks et al., 2023; Gnall et al., 2024; Galluzzi et al., 2024). Instead, no significant MBI effects were observed in the dimensions of “emotional reactions and attentional response to a sensation” (including the not distracting and not worrying subscales) and “attention regulation,” while general increases in such dimensions following mindfulness training were observed in other studies (e.g., de Jong et al., 2016; Fissler et al., 2016; Loucks et al., 2023; Gnall et al., 2024). The reason for the lack of differences in our study is unclear. One possible explanation could be related to the notion that our sample was composed of healthy subjects whereas the other studies focused on patients, or focused on specific MBIs such as MBCT (de Jong et al., 2016; Fissler et al., 2016). However, more research is needed to better explore this issue.

Furthermore, we did not find an effect of time on the “noticing” dimension of IA. Note, however, that, aside from a few exceptions (e.g., Price et al., 2019; Galluzzi et al., 2024), several studies did not find any difference on this dimension following mindfulness training (Bornemann et al., 2015; de Jong et al., 2016; Fissler et al., 2016; Lima-Araujo et al., 2022; D’Antoni et al., 2022; Ugarte Pérez et al., 2023; Feruglio et al., 2023; Gnall et al., 2024). Also, some authors have suggested that mixed results observed in early studies that predominantly used questionnaires and objective measures of interoceptive accuracy, which is a dimension close to MAIA’s “noticing” bodily signals, could be attributed to the low extent with which mind-body practices in general and mindfulness practices in particular exert an effect on this dimension (e.g., Bornemann et al., 2015). Similarly, in line with several studies (e.g., de Jong et al., 2016; Matiz et al., 2020a; D’Antoni et al., 2022; Feruglio et al., 2023), we did not find any MBI effect on the “trust” scores of IA. Some other studies observed instead MBI effects on this dimension, but they were focused on different trainings, such as MBCT (Fissler et al., 2016), or included clinical samples (Fissler et al., 2016; Price et al., 2019). In the current study, the lack of an effect on the “trust” dimension (as well as on “attention regulation”) may be attributed to the small sample size: the average scores at baseline and study completion for these outcomes do suggest a pattern of improvement in response to MTT that may have been detected if the sample size had been larger.

As previously reported, we also observed that, within the MTT group, increases in MAIA self-regulation scores (a component of the MAIA dimension of “awareness of mind-body integration”) were associated with decreases in depression and total emotional distress scores, as measured with the HADS. Although this result should be interpreted with caution due to the small magnitude of the association and the lack of correction for multiple analyses, it can be paired with findings from other studies that showed some dimensions of IA were related to clinical outcomes. For instance, increases in not distracting were found to be associated with reduction in depression severity (de Jong et al., 2016), attention regulation and trusting were found to be associated with increased decentering, which, in turn, was associated with decreased depressive symptoms (Fissler et al., 2016), and increases in MAIA total scores were associated with reduced anxiety (Lima-Araujo et al., 2022). These studies generally suggest that the effects of MBIs on wellbeing may stem from a reduction in experiential avoidance and an enhanced relationship with interoceptive cues, which could promote more adaptive responses to bodily sensations. The difference between the IA dimensions associated with clinical outcomes in these studies and in the present one (“self-regulation”) could again be explained in terms of patient populations, trainings under investigation, outcomes, and measurement instruments. The lack of significance in the association between the changes in the other MAIA dimensions and those in the component/total distress scores may be linked to the small sample size and to the fact that the changes observed in these variables were non-significant.

Overall, this study contributes to the growing body of evidence supporting the potential clinical benefits of mindfulness practice (Creswell, 2017), by focusing specifically on the impact of an MTT program on IA and emotional distress. The findings suggest that enhancing self-regulation through mindfulness training may offer a valuable tool for managing and alleviating mental health symptoms, even in those who will lead MBIs. This is a crucial issue, considering the importance of teacher’s personal embodiment of mindfulness as an effective aspect of the delivery of MBIs and favoring participant outcomes (Crane et al., 2010; Crane et al., 2012; Griffith et al., 2021). Indeed, one study on school-teachers delivering to their students their first MBI after MTT showed that baseline teachers’ stress affected the quality of their implementation of the MBI (Braun et al., 2024).

### 4.1 Strengths of the study

Our study has several strengths. First, the study focuses on individuals training to become mindfulness teachers, a sample that has not yet been thoroughly investigated in the literature. Second, the inclusion of a control group enables a comparison between individuals undergoing the MTT program and those not receiving any mindfulness training, providing stronger evidence for the potential benefits of the intervention. Third, we have explored IA through the use of the MAIA, a well validated questionnaire that allows for a comprehensive assessment of various aspects of IA. Finally, by investigating the relationship between IA and emotional distress, our study confirms and extends previous evidence suggesting the importance of the focus of various aspects of IA, particularly in self-regulation, in the management of emotional wellbeing in both clinical and non-clinical populations.

### 4.2 Limitations of the study

Several limitations should also be considered when interpreting the results of our study. First, MTT group participants were self-selected (and primarily composed of medical doctors and psychologists), potentially leading to a sample that is more motivated and inclined toward mindfulness practices. Additionally, although efforts were made to match the control group participants with the MTT group based on gender, age, and educational background, other factors have not been adequately controlled. Also, only a sub-sample of participants were able to identify an appropriate matched control. The sample size, particularly for the control group, is relatively small, which may limit the generalizability of our findings and may have overshadowed some potential MBI effects. Furthermore, the reliance on self-report questionnaires to measure IA and emotional distress may be subject to response bias and social desirability effects; in particular, self-reports of IA have not consistently been confirmed by performance indexes during interoceptive tasks (e.g., Rominger and Schwerdtfeger, 2024). Finally, the study does not include a follow-up assessment, making it difficult to determine the sustained impact of the MTT program on IA and emotional distress over a longer period of time. Future studies employing randomized controlled designs, active or wait-list control groups, larger samples, objective measurements of IA and distress, as well as follow-up assessments are therefore recommended.

### 4.3 Conclusion

In conclusion, this study adds to the growing body of evidence supporting the positive impact of mindfulness-based interventions on IA and represents the first study assessing such an impact in future mindfulness teachers. The findings suggest that, in a non-clinical population of trainee MBI teachers, mindfulness training can enhance the ability to attend to, and to regulate and interpret bodily signals, potentially fostering a deeper mind-body connection. This may benefit the health of mindfulness teachers and help them be more effective with their mindfulness training participants. Further research is needed to confirm these findings, to better explore the mechanisms underlying these effects, especially over longer periods of time, and to investigate the short- and long-term impact of mindfulness training on IA and mental health in comparison with active comparators.

## Data Availability

The raw data supporting the conclusions of this article will be made available by the authors, without undue reservation.

## In memoriam

This article is dedicated to our friend and colleague Luca Iani, who recently passed away, with whom we had begun to think about the possibility of carrying out this study.
